# Polymeric Nano Drug Delivery Systems for Overcoming Tumor Microenvironment-Mediated Drug Resistance

**DOI:** 10.3390/pharmaceutics18060674

**Published:** 2026-05-29

**Authors:** Yonggyu Kang, Jeongeun Kim, Jisu Park, Subin Lee, Youngjin An, Kwang Suk Lim, Hyun-Ouk Kim

**Affiliations:** 1 Division of Chemical Engineering and Bioengineering, College of Art, Culture and Engineering, Kangwon National University, Chuncheon-si 24341, Gangwon-do, Republic of Korea; kangyg0818@kangwon.ac.kr (Y.K.); 202112527@kangwon.ac.kr (J.K.); qkrwltn0905@kangwon.ac.kr (J.P.); isubin0903@kangwon.ac.kr (S.L.); ayj00306@kangwon.ac.kr (Y.A.); 2 Department of Smart Health Science and Technology, Kangwon National University, Chuncheon-si 24341, Gangwon-do, Republic of Korea; 3 Institute of Fermentation of Brewing, Kangwon National University, Chuncheon-si 24341, Gangwon-do, Republic of Korea

**Keywords:** cancer nanomedicine, drug resistance, polymeric nanoparticle, stimuli-responsive drug delivery, tumor microenvironment

## Abstract

The tumor microenvironment (TME) acts as a major barrier to effective drug delivery and contributes to drug resistance in solid tumors. Hypoxia, acidosis, and elevated interstitial fluid pressure limit drug penetration, while cancer-associated fibroblasts and immunosuppressive cells promote survival signaling, drug efflux, and metabolic adaptation. Polymeric drug delivery systems offer a promising strategy to address these barriers because their structures can be precisely engineered and designed to respond to TME-specific stimuli. These properties enable controlled drug release at tumor sites and help improve therapeutic efficacy while reducing systemic limitations. This review discusses how physicochemical and cellular components of the TME contribute to drug resistance and how polymeric nanomedicines can be designed to overcome these barriers. In addition, it examines key challenges that limit clinical translation, including tumor heterogeneity, variable enhanced permeability and retention effects, manufacturing scalability, and regulatory requirements. Finally, this review highlights the future direction of polymer nanomedicine and focuses specifically on developing rational material design, enhancing preclinical models, and developing clinically appropriate strategies to combat TME-mediated drug resistance.

## 1. Introduction

Cancer remains one of the most serious global health burdens, with nearly 20 million new cases and 10 million deaths reported each year [1]. Although surgery, chemotherapy, radiotherapy, targeted therapy, and immunotherapy have improved patient outcomes, many tumors eventually become less responsive to treatment. Drug resistance after an initial therapeutic response is therefore a major cause of relapse and treatment failure. This problem is recognized in several cancers. For example, resistance from overexpression of MDR1/P-glycoprotein (P-GP) is frequently observed in breast cancer patients receiving doxorubicin-based chemotherapy [2]. In pancreatic cancer, gemcitabine resistance is a major factor in treatment failure [3]. In addition, immune checkpoint inhibitors have a response rate of 20–30% in most solid cancers [4]. Therefore, predicting treatment responses and overcoming resistance are important clinical challenges. A major factor in such drug resistance is the tumor microenvironment (TME).

TME does not consist solely of tumor cells. It is a complex microenvironment in which diverse cells, such as immune cells, fibroblasts, and vascular endothelial cells, interact with extracellular matrix (ECM) and soluble factors [5]. Physical and chemical properties such as hypoxia, acidification, and high interstitial fluid pressure (IFP) significantly inhibit the therapeutic agent from reaching the tumor site. However, many cytotoxic anticancer drugs currently in use do not fully reflect these complex properties of TME. It non-selectively attacks normal and tumor cells, causing serious side effects such as bone marrow suppression, gastrointestinal toxicity, and nephrotoxicity [6]. In addition, existing anticancer drugs have limitations in overcoming resistance at the molecular level. For example, doxorubicin is discharged out of the cell by overexpression of P-gp [7]. Cisplatin is detoxified by increased levels of glutathione (GSH) in cells and increased DNA repair enzyme activity [8]. The reduced efficacy of gemcitabine is attributed to the inactivation by cytidine deaminase and the decreased expression of equilibrative nucleoside transporter 1 (ENT1) [9]. This combination of molecular-level resistance and TME-derived physicochemical barriers reduces the effectiveness of conventional treatments. Therefore, it is necessary to develop a novel drug delivery method that can precisely represent the characteristics of TME.

Polymeric drug delivery systems (PDDSs) have been developed to address some of these limitations. Their composition, molecular weight, hydrophobicity, degradation rate, and surface chemistry can be adjusted during design. This facilitates enhanced regulation of drug loading, circulatory dynamics, tumor localization, and release kinetics. Biodegradable polymers, including PLGA, PLA, and PCL, are extensively utilized due to their capacity for regulated drug release and recognized biomedical significance [10]. In addition, amphiphilic poly-N-vinylpyrrolidone (PVP) derivatives have been explored as micellar and nanoparticulate carriers, in which hydrophilic PVP segments provide a biocompatible corona and hydrophobic segments enable self-assembly and loading of poorly water-soluble anticancer cargos [11,12]. Furthermore, polymeric carriers can be modified by targeting ligands or shielding groups to improve tumor localization and cellular uptake. They can also be designed to respond to TME-related signals such as acidic pH, redox gradients, reactive oxygen species, and hypoxia [13]. Through these features, PDDSs may improve drug availability at tumor sites while reducing unnecessary exposure to normal tissues.

This review examines the cellular and molecular mechanisms of drug resistance through TME, comparative evaluation with polymer nanoplatforms to overcome them, TME’s reactive design strategy, current status of treatment application and clinical translation (Section 6), future challenges, and development direction of precision nanomedicine. In this review, the term “polymeric nanosystems” is used to describe broad polymer-based nanoscale delivery platforms, including nanoparticles, micelles, polymersomes, nanogels, dendrimers, polymer–drug conjugates, and hybrid systems. The term “polymeric nanomedicines” is used when these systems are discussed in the context of therapeutic application or clinical translation.

## 2. Tumor Microenvironment and Its Role in Drug Resistance

### 2.1. Key Components of the Tumor Microenvironment

**Figure 1:**
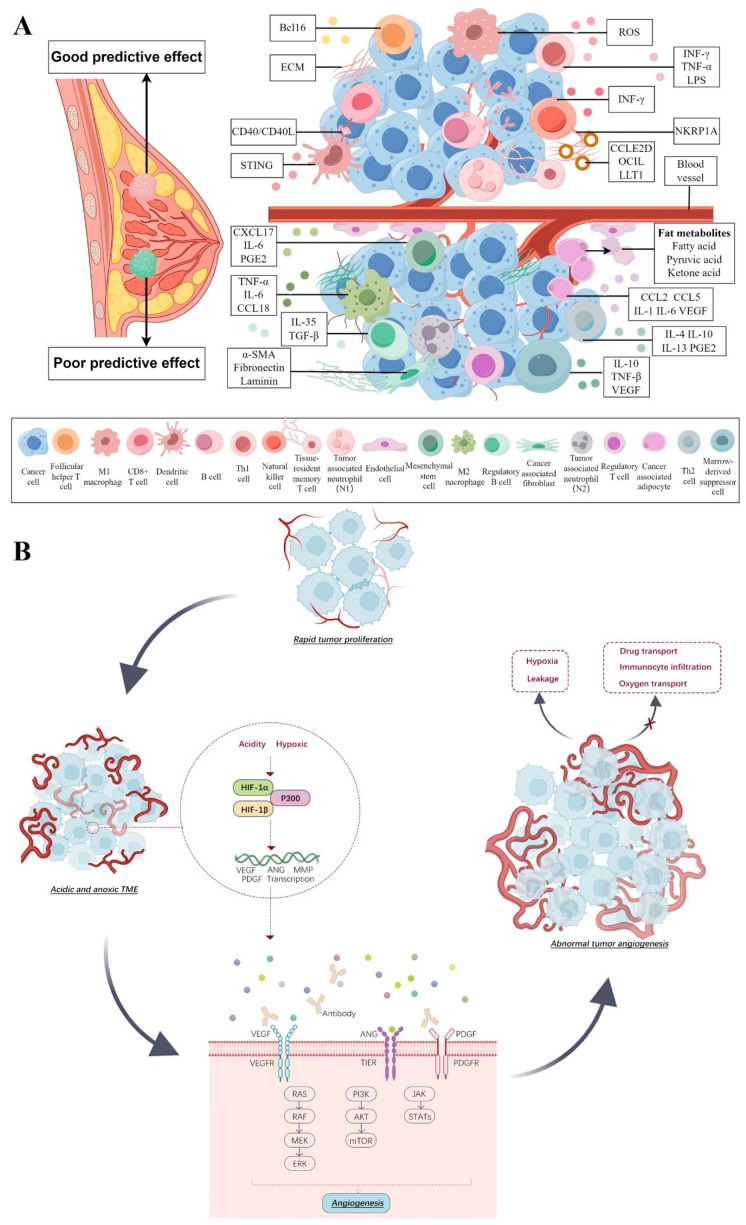
**Tumor microenvironmental heterogeneity and physicochemical barriers associated with therapeutic resistance.** (**A**) Cellular heterogeneity of the tumor microenvironment and its association with therapeutic outcome. (**B**) Physicochemical barriers of the tumor microenvironment, including abnormal vasculature, hypoxia, acidosis, and impaired drug transport. The curved arrows indicate a feedback loop among tumor proliferation, hypoxic/acidic TME formation, angiogenic signaling, and abnormal angiogenesis. Colored circles represent soluble mediators, metabolites, and angiogenic factors within the TME. The overlapping tumor cells and tortuous vessels depict the dense spatial coupling of tumor growth and abnormal vasculature. Adapted from Dou et al., Front. Immunol. 15, 1368687 (2024) [22], licensed under CC BY 4.0. Adapted from Wang et al., Front. Immunol. 15, 1472772 (2024) [23], licensed under CC BY 4.0.

The tumor microenvironment (TME), which is a complex environment that surrounds tumor cells (Figure 1A), is a key factor in determining how tumors grow and how well treatments work. The TME can be broadly divided into cellular and non-cellular components. Cancer-associated fibroblasts (CAFs), immune cells, endothelial cells, pericytes, and substrate cells are all types of cells. Extracellular matrix (ECM), blood vessels, cytokines, chemokines, and growth factors are all non-cellular components [14]. CAF is a major regulatory components of the TME. It contributes to tumor progression and the formation of therapeutic resistance through ECM reconstruction, promoting angiogenesis and immune regulation. Recently, functionally heterogeneous subtypes such as myofibroblastic CAF (myCAF), inflammatory CAF (iCAF), and antigen-presenting CAF (apCAF) have been reported [15]. ECM is also more than just a support. It interacts with immune cells and acts as a functional factor that regulates cancer progression and immunotherapy response [16]. Immunosuppressive cells, including M2-like tumor-associated macrophages (M2-like TAMs), bone myeloid-derived suppressor cells (MDSCs), and regulatory T cells (Tregs), predominate in the tumor microenvironment (TME). Moreover, tumor-associated endothelial cells generate aberrant vascular networks. This establishes a tumor-protective microenvironment characterized by the interaction of temperamental, immunological, and vascular components [17].

### 2.2. Physicochemical Barriers Including Hypoxia, Acidity, and High Interstitial Pressure

The TME consists of cellular and stromal elements, together with physicochemical features such as hypoxia, acidosis, and elevated interstitial fluid pressure (IFP) (Figure 1B). These conditions result from rapid tumor growth, abnormal vascular structures, inadequate lymphatic drainage, and sustained substrate reconstruction. These factors do not act independently but interact with one another to further reinforce therapeutic resistance. Hypoxia is a common microenvironmental feature in solid cancers. These factors activate hypoxia-inducible factor-1α (HIF-1α) signals, which induce angiogenesis, metabolic reprogramming, immune avoidance, ECM reconstruction, and enhanced survival signals [18,19]. The acidified extracellular environment (pH approximately 6.5) reduces drug invasion, increases infiltration, and weakens immune response [20]. Elevated IFP acts as a barrier to drug penetration in tumor tissues. It is driven by vascular leakage, impaired lymphatic drainage, dense ECM deposition, and stromal compression, thereby restricting the uniform distribution of therapeutic agents [21].

### 2.3. Cellular Contributors Such as Cancer Associated Fibroblasts and Immune Suppressive Cells

**Figure 2:**
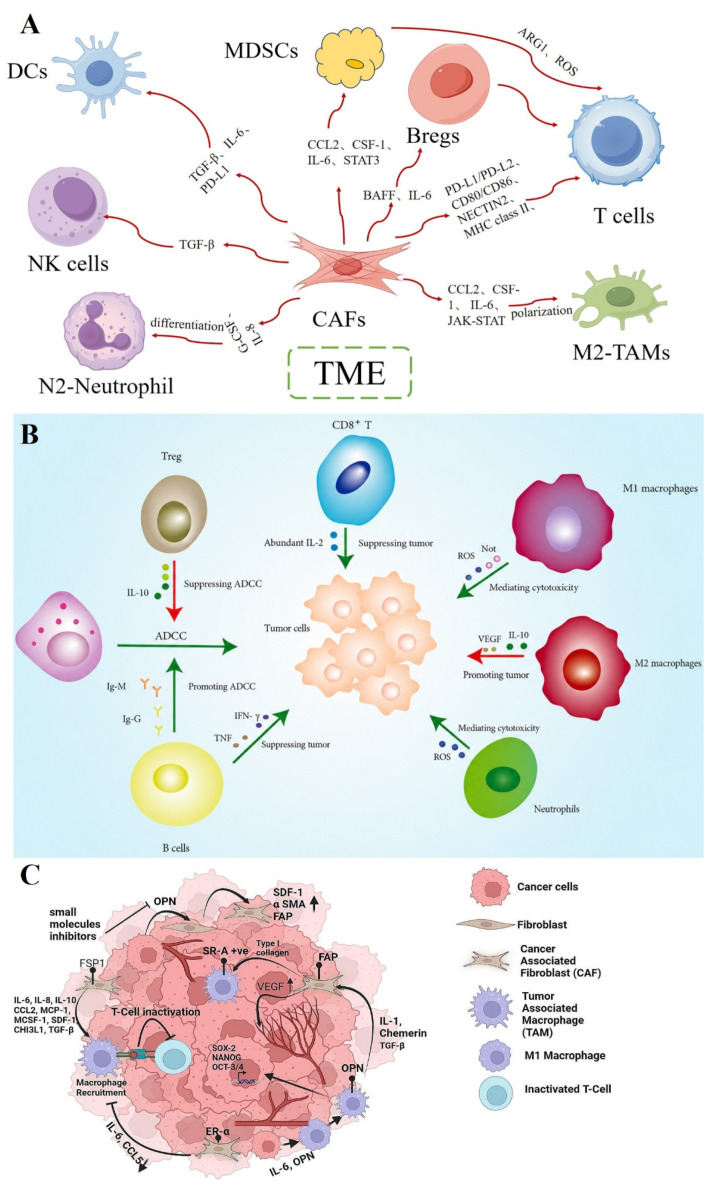
**Immunosuppressive cellular crosstalk within the tumor microenvironment.** (**A**) CAF-centered crosstalk with immune-suppressive cells in the tumor microenvironment. (**B**) Protumor and antitumor functions of major immune cell subsets within the tumor microenvironment. (**C**) Crosstalk between CAFs and M2-like tumor-associated macrophages in shaping an immunosuppressive tumor microenvironment and promoting resistance to immunotherapy in breast cancer. Adapted from Luo et al., Front. Immunol. 16, 1617662 (2025) [27], licensed under CC BY 4.0, and Yu, J. et al., Front. Immunol. 16, 1517959 (2025) [28], licensed under CC BY 4.0. Adapted from Kundu et al., *Mol. Cancer* 23, 92 (2024) [29], licensed under CC BY 4.0.

Cellular contributing factors of TME play an important role in the formation and maintenance of drug resistance. Among the TME components discussed in Section 2.1, CAFs and immunosuppressive immune cells maintain continuous crosstalk with tumor cells and establish a protective microenvironment (Figure 2A,B). CAFs secrete transforming growth factor-β (TGF-β), interleukin-6 (IL-6), and CXCL12, thereby supporting tumor survival and therapeutic resistance. CAFs thereby promote tumor growth, invasion, stemness, and resistance to apoptosis [24]. It also sends survival evasion signals via exosome cargo. CAFs promote ECM synthesis and remodeling, which increases tissue stiffness and vascular compression, limiting medication penetration. They contribute to different treatment resistances via functional subtypes such as myCAF and iCAF [25]. From the perspective of immunosuppressive cells, M2-like TAMs, MDSCs, and Tregs suppress the activity of cytotoxic T cells and natural killer cells (NK cells). An interleukin-10 (IL-10) and TGF-β promote immune avoidance [26]. These cell populations are also closely related to angiogenesis, tumor stemness, metabolic reorganization, and recurrence. It contributes to enhanced drug resistance.

### 2.4. Impact of the Tumor Microenvironment on Therapeutic Resistance

The TME is a regulatory nexus that dictates therapeutic outcomes through an interconnected network of cellular and physicochemical factors (Figure 2C). Rather than acting in isolation, these components converge to form a multifaceted foundation for resistance, encompassing inhibited drug delivery, activated survival signaling, and metabolic adaptation. While physicochemical barriers physically impede the arrival of therapeutic agents at their target, cellular components activate molecular-level programs such as drug efflux and oxidative stress responses. These pathways promote cancer cell survival and recurrence. Such an integrated perspective suggests that a resistant tumor treatment strategy should not only aim at a single mechanism but also coordinate the multi-layered structure of TME at the same time.

## 3. Mechanisms of Tumor Microenvironment Mediated Drug Resistance

### 3.1. Impaired Drug Delivery and Limited Tumor Penetration

**Figure 3:**
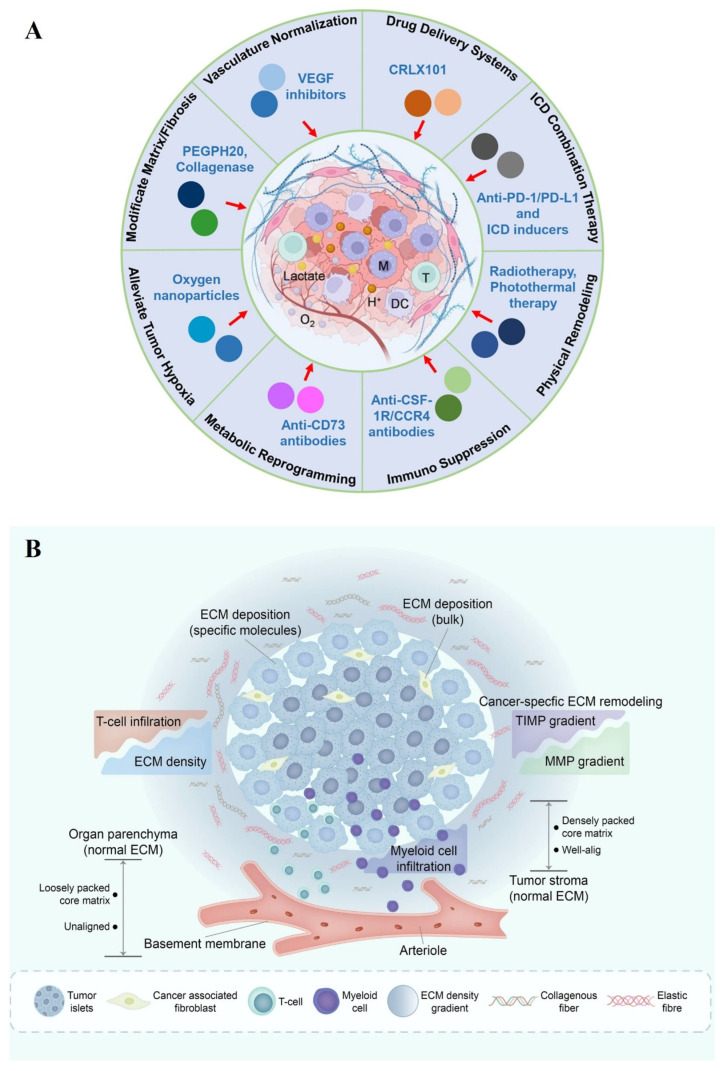
**Tumor microenvironmental barriers and stromal remodeling associated with therapeutic resistance.** (**A**) Major tumor microenvironmental barriers and representative strategies to improve drug delivery and therapeutic efficacy. Red arrows indicate the direction of therapeutic intervention toward the tumor site, and colored circles schematically denote representative therapeutic agents or strategies for each barrier category. (**B**) Extracellular matrix remodeling and stromal organization that restrict immune-cell infiltration and intratumoral transport. Adapted from Zhang et al., Front. Immunol. 16, 1672601 (2025) [34], licensed under CC BY 4.0; and Du et al., Front. Immunol. 14, 1340634 (2023) [35], licensed under CC BY 4.0.

The early stage of drug resistance via TME is a transport barrier in which the drug does not reach the tumor sufficiently and uniformly (Figure 3). The physicochemical properties of the TME, as discussed in Section 2, can be examined step by step to understand how they influence the transportation process. The first is the vascular transport stage, in which abnormal tumor vessels lead to uneven blood flow, vessel compression, and partial obstruction. These changes reduce the amount of drug that reaches the tumor vasculature [30]. The second is the interstitial diffusion stage, during which drugs must move from the tumor vessels into the surrounding tumor tissue. The reconstructed ECM and CAF-derived fibrosis physically narrow the diffusion path and cause a shearing effect. High IFPs inhibit convective transport and limit intrahepatic movement of drugs [31]. Third, it is the interaction stage with cells and substrates. Non-specific binding to ECM components can create a binding site barrier that sequesters drugs within the tumor stroma, thereby reducing the effective drug concentration available for tumor penetration and cellular uptake [32,33]. As a result, anticancer drugs are limited to the periphery of the blood vessels. Only low concentrations reach the center of the tumor or the site where fibrosis has progressed, allowing survival and recurrence of residual cells. Such inadequate drug exposure is more than just a result. Repeated exposure to low concentrations serves as an initial trigger for resistance that activates survival signals and induces molecular-level resistance programs.

### 3.2. Activation of Survival Signaling and Inhibition of Apoptosis

**Figure 4:**
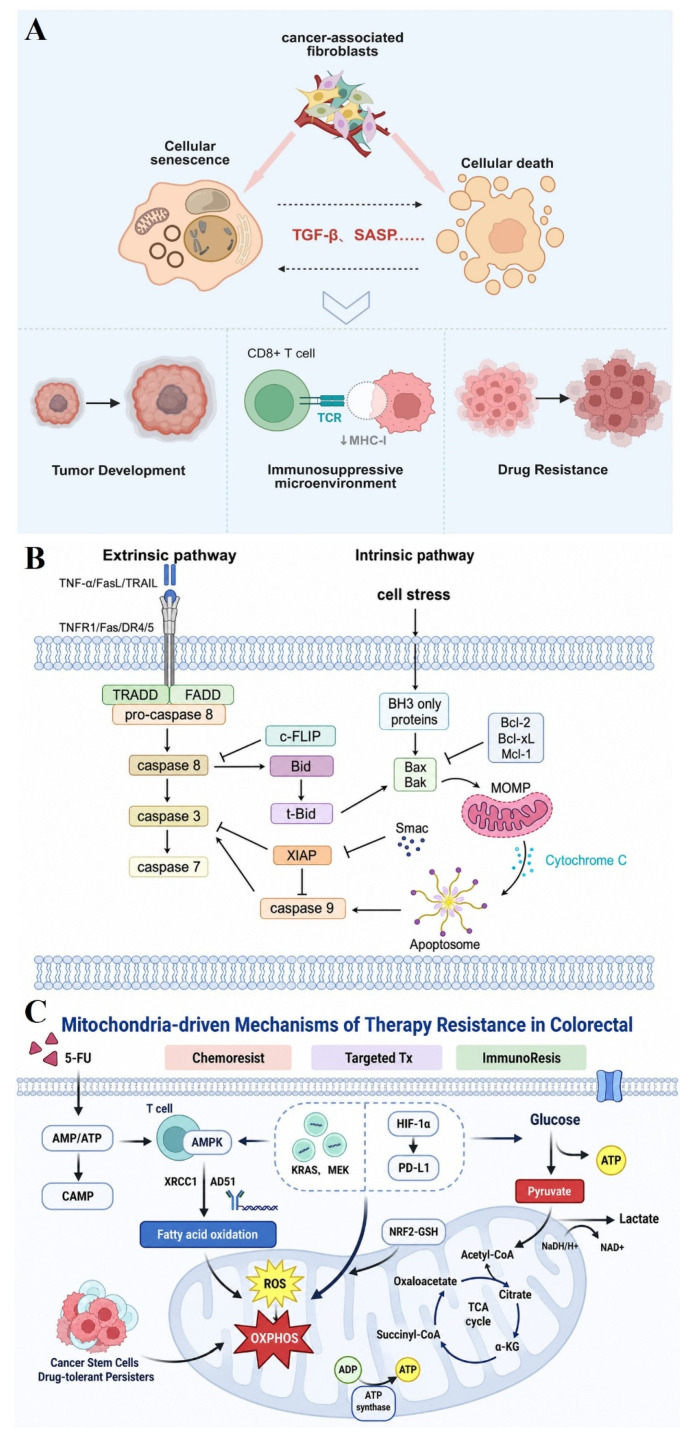
**Cellular and metabolic mechanisms underlying tumor therapeutic resistance.** (**A**) CAF-mediated regulation of cellular senescence and cell death, contributing to an immunosuppressive microenvironment and drug resistance. The arrows indicate CAF-driven regulatory interactions among cellular senescence, cell death, tumor development, immune suppression, and drug resistance. (**B**) Schematic overview of the extrinsic and intrinsic apoptotic pathways and their suppression by anti-apoptotic regulators. The arrows and inhibitory lines indicate activation and suppression steps within apoptotic signaling cascades. (**C**) Integrated metabolic adaptations associated with drug efflux, extracellular acidosis, oxidative stress regulation, immune modulation, and apoptosis evasion in resistant tumor cells. The arrows indicate metabolic fluxes and signaling interactions that support therapy-resistant tumor phenotypes. Adapted from Ruan et al., Front. Immunol. 16, 1635771 (2025) [45], licensed under CC BY 4.0; and Wang et al., Cell Death Discov. 11, 93 (2025) [46], licensed under CC BY 4.0. Adapted from Qiu et al., Cell Death Discov. 11, 375 (2025) [47], licensed under CC BY 4.0.

TME-mediated drug resistance progresses by enhancing survival signals inside cancer cells and inhibiting apoptosis, overcoming transport barriers. Hypoxia, inflammatory cytokines, growth factors, metabolic stress, and ECM-derived signals activate major survival pathways. This includes the phosphoinositide 3-kinase (PI3K)/AKT/mammalian target of rapamycin (mTOR), Janus kinase (JAK)/signal transducer and activator of transcription 3 (STAT3), nuclear factor kappa B (NF-κB), and mitogen-activated protein kinase (MAPK) pathways [19,36]. These pathways are driven by TME-derived signals. For example, IL-6 activates the JAK/STAT3 pathway. TGF-β activates the SMAD and non-SMAD pathways. Hypoxia-derived ROS activates NF-κB [37]. The activated pathway increases the expression of anti-apoptotic factors such as the B-cell lymphoma 2 (BCL-2) family and survivin. This weakens therapy-induced apoptosis [38]. This process also directly involves the cellular components of TME. CAFs activate bypass signaling and anti-apoptotic pathways through IL-6, CXCL12, TGF-β, and exosome cargo (Figure 4A,B). M2-like TAMs also regulate the PI3K/AKT, JAK/STAT, and MAPK pathways, thereby promoting cancer cell survival, stemness, epithelial–mesenchymal transition (EMT), and resistance to apoptosis [39]. Ultimately, the continuous activation of survival signals and the inhibition of apoptosis act as key mechanisms through which the TME prolongs treatment resistance. To enhance the therapeutic effect, it is necessary to regulate not only the cancer cells themselves but also the interactions within the TME.

### 3.3. Drug Efflux, Metabolic Adaptation, and Oxidative Stress Response

TME-mediated drug resistance continues to operate at the molecular level even after the therapeutic agents reach the tumor cells. This phenomenon is explained by three complementary mechanisms. First, it is drug efflux. Hypoxic microenvironments and various TME-derived signals increase the expression and activity of ATP-binding cassette (ABC) transporters. This includes P-gp, ABCC1/multidrug resistance protein 1 (MRP1), and ABCG2/breast cancer resistance protein (BCRP) [40]. The activated transporter expels the anticancer drug out of the cell. As a result, cancer cells acquire a multidrug resistance (MDR) phenotype while maintaining a low intracellular drug concentration. Second, it is metabolic adaptation. This is a key mechanism that enables tumor cells to survive under therapeutic stress. Tumor cells and TME constituent cells reprogram their metabolism to adapt to nutrient deprivation, hypoxia, and acidosis. Glucose metabolism (enhanced glycolysis based on the Warburg effect), lipid, and amino acid metabolism are altered [41]. Through this, it secures not only an ATP supply but also biosynthetic precursors and reducing power (NADPH). As a result, DNA damage repair, evasion of apoptosis, maintenance of stemness, and immune evasion are promoted. Third, it is the oxidative stress response. This is an adaptive mechanism to the accumulation of ROS induced during the treatment process. Tumor cells utilize an antioxidant network based on GSH, glutathione peroxidase 4 (GPX4), thioredoxin, and nuclear factor erythroid 2-related factor 2 (NRF2), as well as autophagy-based protective mechanisms. Through these processes, they buffer oxidative damage and evade apoptosis and ferroptosis [42,43]. These three mechanisms do not operate independently. Metabolic reprogramming supplies the ATP needed for the ABC transporter function. The oxidative stress response is linked to efficient metabolic regulation. Therefore, they should be understood as an integrated resistance system [44]. This suggests the need for polymer nanoplatforms that target multiple mechanisms simultaneously rather than a single target inhibition (Figure 4C).

## 4. Polymeric Drug Delivery Systems

### 4.1. Polymeric Nanoparticles and Micelles

**Figure 5:**
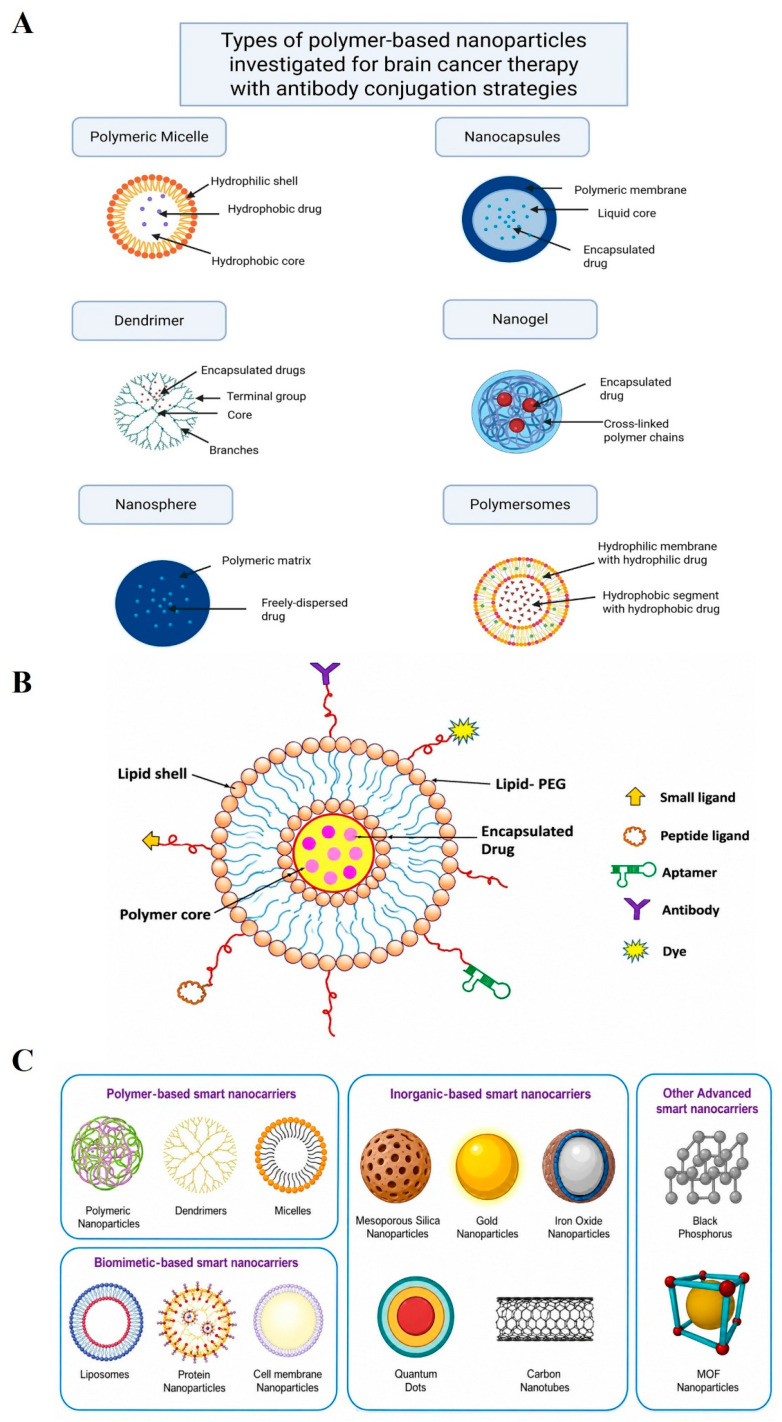
**Representative polymeric nanoplatforms for cancer drug delivery.** (**A**) Structural types of polymer-based nanoparticles, including polymeric micelles, nanocapsules, dendrimers, nanogels, nanospheres, and polymersomes. (**B**) Schematic illustration of a lipid–polymer hybrid nanoparticle with a polymer core, lipid shell, PEGylated surface, and ligand-functionalized exterior for targeted drug delivery. (**C**) Classification of smart nanocarriers, highlighting polymer-based systems together with biomimetic, inorganic, and other advanced nanoplatforms used in cancer therapy. Adapted from Tai et al., Drug Deliv. Transl. Res. 15, 4367–4410 (2025) [65], licensed under CC BY 4.0. Adapted from Gajbhiye et al., Mol. Cancer 22, 160 (2023) [66], licensed under CC BY 4.0. Adapted from Sun et al., Signal Transduct. Target. Ther. 8, 418 (2023) [55], licensed under CC BY 4.0.

Polymeric nanoparticles and polymer micelles are the most widely studied polymer-based drug delivery platforms to overcome drug resistance through TME (Figure 5A). Certain polymeric nanoparticle formulations may inhibit P-gp-mediated recognition and efflux of encapsulated drugs [48], while PEGylation can prolong systemic circulation by reducing protein adsorption and mononuclear phagocyte system-mediated clearance. The enhanced permeability and retention (EPR) effect causes passive tumor accumulation [49], although dependence on the EPR effect requires caution in clinical trial translation. The effects of EPR are observed relatively consistently in preclinical animal models. However, in human tumors, vascular permeability fluctuations are large between patients, tumors, and intra-tumor sites [50], so EPR-based passive accumulation alone is difficult to ensure clinical consistency. This is why active targeting and stimulus-reactive design are complementarily required. Typical biodegradable polymers include poly(lactic-co-glycolic acid) (PLGA), poly(lactic acid) (PLA), poly(caprolactone) (PCL), and amphiphilic poly-N-vinylpyrrolidone (PVP). Polymeric micelles are formed by self-organization of amphiphilic block copolymers. It captures insoluble drugs in a minority nucleus and enhances stability and circulation through a hydrophilic outer shell [51]. When disulfide binding is introduced into micelles, it is decomposed in response to high GSH concentrations in resistant cancer cells. As a result, drugs can be released quickly in cells [52]. By such characteristics, selective drug release is possible even in an environment in which the leakage pump is operated.

### 4.2. Polymersomes, Nanogels, and Dendrimer-Based Systems

Advanced platforms, by deepening the design principles of nanoparticles and micelles, include polymersomes, nanogels, and dendrimers. The polymersome is based on an amphiphilic block copolymer double-membrane structure. Hydrophilic agents (internal aqueous space) and hydrophobic agents (in the double membrane) can be loaded simultaneously. By responding to pH, redox conditions, hypoxia, or light, these systems can achieve controlled drug release within the TME [53,54]. Nanogels possess a three-dimensional cross-linked network structure that enables high drug-loading capacity. capable of expanding or contracting according to TME-specific stimuli and controlling the release of drugs [55]. Dendrimers have regular branched structures and numerous surface functional groups. The size and shape can be precisely controlled in the synthesis stage. In addition, treatment, targeting, and diagnostic functions can be integrated into a single structure depending on the design [55]. The structural diversity and stimulus responsiveness of these platforms enable them to effectively respond to complex resistance mechanisms. Nanogels can help combat TME-mediated MDR by combining various therapeutic drugs and functional moieties.

### 4.3. Polymeric Drug Conjugates and Hybrid Polymeric Nanocarriers

Polymer-drug conjugates and hybrid nanocarriers are strategies for covalently binding or combining macromolecules and drugs or lipids. These strategies will overcome the limitations of the single platform. A polymer drug complex is a method of covalently binding a drug to a polymer skeleton. The linker used at this time can be designed to respond to TME-specific signals such as pH, GSH concentration, enzyme activity, etc. [56]. When such bonds are selectively cleaved within the tumor, the drug is activated. As a result, the prodrug state is maintained in normal tissue, and selective activation is realized, which converts to an active type only within the tumor. Typical examples include N-(2-hydroxypropyl) methacrylamide (HPMA)-doxorubicin conjugates (PK1, PK2) and paclitaxel poliglumex (opaxio) evaluated in clinical stages [57,58]. Lipid–polymer hybrid nanoparticles (LPNs) are representative hybrid nanocarriers (Figure 5B). LPNs combine the biocompatibility and cellular affinity of lipids with the structural stability of polymeric nanoparticles. This design can reduce the limitations of single-component systems while improving safety and therapeutic efficacy [59]. As such, the polymer drug complex and hybrid nanocarriers combine structural multifunctionality and TME reactivity. This platform can improve drug activation and intracellular delivery in resistant tumors.

### 4.4. Advantages of Polymeric Platforms for Overcoming Drug Resistance

Each platform has its own strengths and limitations, and on which resistance axis it exerts its advantage is different (Figure 5C). First, drug loading capacity and tumor penetration efficiency. Second, responsiveness design accuracy of TME. Third, reproducibility of manufacturing and ease of large-scale production. Fourth, the maturity of clinical translation. Polymer nanoparticles and polymer micelles are the most mature platforms. The manufacturing process is well established, and the reproducibility between batches is relatively excellent. For clinical translation, several polymeric micelle formulations, including Genexol-PM and NK105, have been evaluated clinically [60]. However, their drug-loading capacity is mainly suited for single hydrophobic compounds. Since it tends to rely on passive targeting, additional active targeting and stimulus-reactive functions improve the efficiency of resistance overcoming. Polymersomes are most advantageous for simultaneous loading of hydrophilic and hydrophobic agents owing to their double membrane structure. The accuracy of the multi-stimulus reactive design is high. However, it is difficult to secure manufacturing reproducibility due to the high sensitivity to process parameters in self-organization of block copolymers [61]. To date, clinical cases have been limited, and most are in the preclinical stage. A relatively large size (100–200 nm) may also function as an infiltration limit in tumors with dense ECM. Nanogels show the highest drug load thanks to the three-dimensional cross-linking network. It is particularly advantageous for loading water-soluble drugs and biomolecules (siRNAs and proteins) [62], but it is disadvantageous for infiltration into deep tumors due to its increased size in an expanded state. It tends to show a relative advantage in intramucosal delivery and local administration. Dendrimers are advantageous for precise molecular-level synthetic control and penetration into deep tumors in a small size of a few nanometers. Can integrate treatment, targeting, and diagnostic functions into a single structure [63]. However, the higher the production, the more complex the synthesis process becomes and the higher the cost. Because cationic surfaces may induce toxicity, appropriate surface modification is required. The case of clinical translation is limited to some PAMAM systems. A polymer drug conjugate is an activation strategy similar to a prodrug and has a relatively long track record of clinical translation. Several clinical trials, including PK1, PK2, and Opaxio, were conducted [57,58], although kinetic control of activation based on linker design is difficult. Compared to low-molecular drugs, the amount of loading is limited, and several tests have pointed out results that do not meet expectations. Lipid–polymer hybrid nanoparticles (LPNs) are an emerging platform that combines the structural stability of macromolecules with the biocompatibility of lipids. It has excellent loading efficiency and cell affinity. However, it is difficult to scale up the process and control the quality of the production due to the double structure of polymer and lipid [64]. Overall, the most mature platforms for clinical applications at this time are polymer micelles and polymer drug complexes. Polymersomes, nanogels, dendrimers, and LPNs exhibit superiority in functional elaboration. However, the complexity of manufacturing and quality control remains a major barrier to clinical translation. In addition, drug loading capacity and tumor penetration efficiency often show a trade-off relationship. For example, nanogels provide a high loading capacity but are disadvantageous for penetration, and dendrimers are advantageous for penetration, but their loading capacity is limited. Based on this comparison, the following Section 5 specifically discusses three key design strategies that maximize TME responsiveness.

## 5. Design Strategies for Overcoming Tumor Microenvironment Mediated Drug Resistance

### 5.1. Stimuli-Responsive Polymeric Nanosystems for pH, Redox, ROS, and Hypoxia

Reactive polymer nanosystems are delivery platforms designed to selectively release drugs in response to specific stimuli of TME. Representatively, four stimuli are utilized (Figure 4A). A pH-reactive polymer induces the decomposition and structural change in polymer bonds in a weak acidic tumor microenvironment (pH approximately 6.5) or endosome/lysosome (pH 5.0 to 4.0). This releases the drug [67]. Typical acid-unstable bonds include hydrazone, acetal, orthoester, and imine bonds. These bonds are hydrolyzed under acidic conditions, causing micelles and nanoparticle dismantling. Redox-reactive macromolecules utilize a high reduction environment in tumor cells. The intracellular GSH concentration is about 10 mM, 100–1000 times higher than the extracellular concentration [68,69]. Therefore, polymer nanoparticles containing disulfide bonds (–S–S–) are selectively decomposed in the cell by thiol-disulfide exchange. As a result, drugs are released. ROS-reactive macromolecules take advantage of the fact that tumor cells have higher ROS concentrations than normal cells due to rapid metabolism and abnormal mitochondrial function [70]. Bonds that are oxidatively cleaved by ROS, such as thioketal, boron ester, allylboronate, selenium, etc., are introduced into the polymer structure. This allows the macromolecules to be selectively decomposed within the tumor tissue, and the drug can be released. Hypoxia-responsive macromolecules have low oxygen conditions (O_2_ partial pressure) and utilize functional groups reduced by nitroreductase overexpressed below 10 mmHg [71]. A reduction reactive group such as Azo bond, 2-nitromidazole (2-NI), and nitrobenzene is introduced. As a result, the conversion from hydrophobic to hydrophilic occurs, inducing the dissolution of micelles and the release of drugs.

The distinct mechanisms, chemical linkages, and biological triggers of these four stimuli-responsive systems are comprehensively summarized in Table 1.

### 5.2. Surface Engineering and Ligand-Mediated Targeting Strategies

**Figure 6:**
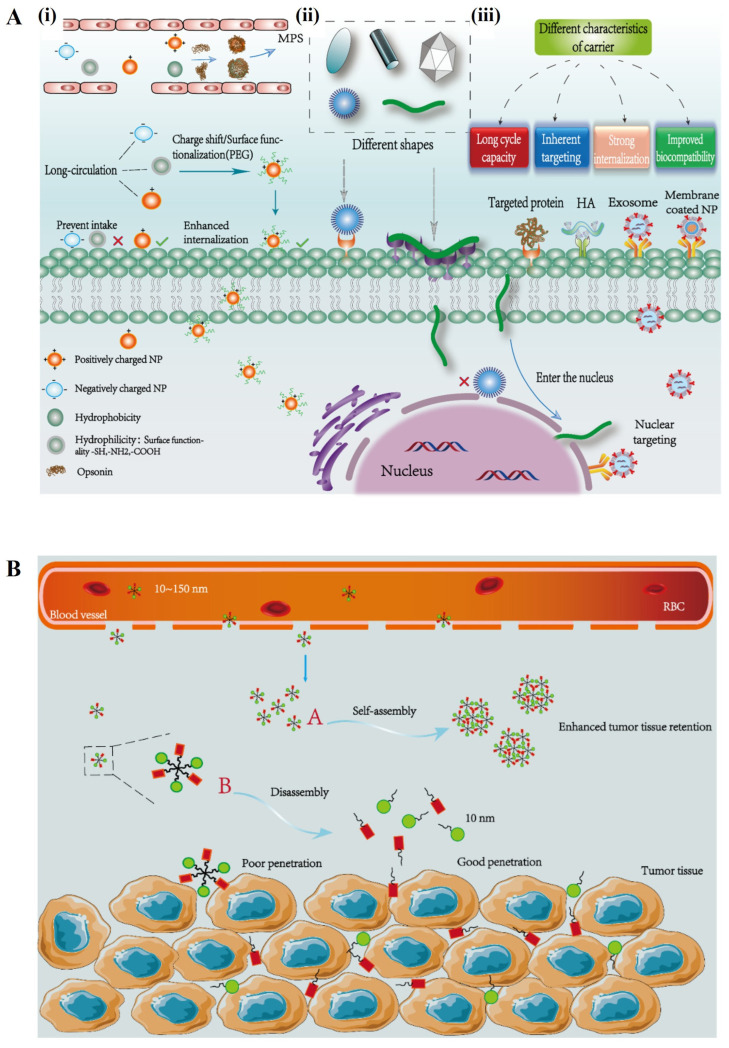
**Surface engineering and ligand mediated targeting strategies** (**A**) Effects of surface properties, shape and intrinsic properties on the targeting of NPs (**i**) Transport of particles with different charge and surface chemical properties in vivo; (**ii**) schematic diagram of cell uptake of NPs with different shapes; vermicular carriers find it easier to enter intracellularly by polyvalent contact with the membrane surface, and they even achieve nuclear targeting; (**iii**) different intrinsic characteristics of the carrier. (**B**) Schematic illustration of the systemic transport and tumor delivery of 10–150 nm nanoparticles. Adapted from Yang et al., Pharmaceutics 14, 1919 (2022) [85], licensed under CC BY 4.0.

Surface modification of polymer nanoparticles is an important strategy to increase selective delivery and cell absorption efficiency to tumor tissue (Figure 6A). A typical example is PEGylation conversion that introduces polyethylene glycol (PEG) to the nanoparticle surface. As a result, a hydrophilic barrier is formed, and adsorption of proteins and removal by macrophages are suppressed. As a result, the circulation time of nanoparticles in the blood is extended [72]. Through this mechanism, nanoparticles may be passively accumulated according to the EPR effect utilizing the tumor’s abnormal vascular structure. However, as discussed in Section 4.1, the effect of EPR is large among patients with human tumors. Therefore, designs that rely solely on PEGylation and passive accumulation have limitations in terms of clinical translation reliability [73,74]. Ligand-mediated targeting is an active targeting strategy that complements these limitations. bind specific ligands to the surface of nanoparticles and selectively to receptors on the surface of tumor cells [75]. Typical ligand-receptor pairs include a folate receptor (FR), transferrin receptor (TfR), RGD peptide integrin αvβ3, iRGD-neuropilin-1 (NRP-1), hyaluronic acid-CD44, and antibody-based targeting (anti-HER2, anti-EGFR) [76,77,78]. Such an approach can improve selective delivery to tumor cells and intracellular invasion. In recent years, a composite surface modification strategy that simultaneously applies PEG and ligand-mediated targeting has been actively studied. These systems are stable in circulation and enhance delivery via target-specific interactions in tumor tissues [79]. Cell membrane coating is also a biomimicry strategy that covers the surface of polymer nanoparticles with cell membranes extracted from red blood cells, white blood cells, platelets, cancer cells, etc. Consequently, immuno-avoidance, homotypic targeting, and biocompatibility can be improved simultaneously [80].

### 5.3. Size, Charge, and Penetration Optimizing Approaches

Nanoparticle size, surface charge, and penetration into tumor tissue are important factors in determining drug delivery efficiency. In general, extremely small particles below 5 to 10 nm are rapidly removed by renal filtration [78]. Large particles larger than 200 nm are easily removed by the mononuclear phagocyte system (MPS). On the other hand, nanoparticles in the range of 10 to 100 nm are easily accumulated in tumor tissue due to the EPR effect (Figure 6B) [77]. It affects interaction with cell membranes transmitted to the surface. Nanoparticles with neutral or weak negative charges have less nonspecific binding to blood proteins and have a longer circulation time. Nanoparticles with positive charges increase their inflow into the cell through electrostatic interaction with the cell membrane. However, highly positively charged nanoparticles can be removed quickly in the blood or cause toxicity [81], and accordingly, charge-conversion nanoparticles have attracted attention in recent years. They maintain neutral or negative charges in the blood and change to positive charges under the tumor’s acidic environment [82,83]. Tumor tissue is limited in diffusion of nanoparticles due to dense ECM and collagen fibers. Three strategies are being used to improve this process. First, it is a strategy to jointly deliver ECM-degrading enzymes such as collagenase and hyaluronidase. Second, it is a strategy to introduce tumor-penetrating peptides such as iRGD onto the surface to induce tumor penetration into the deep. Third, the matrix metalloproteinase (MMP) reactive size conversion system involves the use of reduced nanoparticles. During the circulation stage, the EPR effect is utilized in a large size, and after the tumor is reached, it is switched to a small size to ensure penetration [84].

## 6. Therapeutic Applications and Translational Progress

### 6.1. Polymeric Nanosystems for Chemotherapy Sensitization

**Figure 7:**
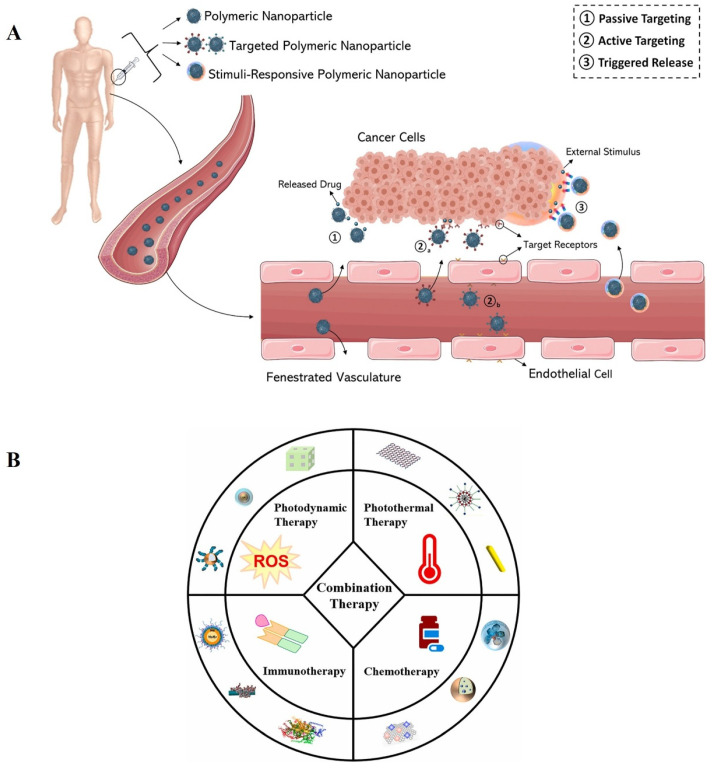
**Polymeric Nanoparticle Delivery Strategies in Tumor Tissue and Therapeutic Applications.** (**A**) polymeric nanoparticles accumulate in tumors through passive targeting via the enhanced permeability and retention effect, active targeting through receptor-mediated interactions, and stimuli-triggered release at the tumor site. These strategies improve local drug accumulation and controlled release in cancer tissues. (**B**) Combination strategies for cancer therapy. Adapted from Gagliardi et al., Front. Pharmacol. 12, 601626 (2021) [91], licensed under CC BY 4.0. Adapted from Liu et al., Nanophotonics 10, 3391–3395 (2021) [92], licensed under CC BY 3.0.

Chemotherapy sensitization refers to a strategy that allows cancer cells to respond more sensitively to anticancer drugs and has a higher therapeutic effect at the same drug concentration (Figure 7) [86]. There are four main categories of sensitization strategies using polymer nanosystems. The first is the increase in tumor accumulation due to drugs. Polymeric nanoparticles are selectively accumulated in tumor tissue through the EPR effect. It is efficiently delivered to the inside of cancer cells through ligand-mediated targeting to increase the concentration of drugs in the tumor [87]. The second is inhibition of drug resistance mechanisms. Polymeric nanosystems can design co-delivery systems that simultaneously deliver anticancer drugs and P-gp inhibitors. Typical P-gp inhibitors include verapamil, tariquidar, and cyclosporin A. The strategy extends drug retention time in cells and increases therapeutic effectiveness [88]. The third is improvement of drug sensitivity through TME adjustment. Using nanosystems that produce or supply oxygen to relieve tumor hypoxia, chemotherapy and radiation can increase effectiveness. Typical examples are perfluorocarbon-based oxygen carriers and MnO_2_-based in situ oxygen production systems [89]. The fourth is a combination of gene therapy and chemotherapy. It uses siRNA or shRNA to suppress the expression of drug resistance-related genes such as MDR1, BCL-2, and survivin. This can reduce anticancer drug resistance in cancer cells [90]. Recently, research has been underway to design drugs and sensitizers to be released selectively only within tumor tissue using various stimulus-reactive polymer nanosystems. Through this, attempts to further improve treatment efficiency are active [67].

### 6.2. Combination Strategies with Immunotherapy, Phototherapy, and Gene Therapy

**Figure 8:**
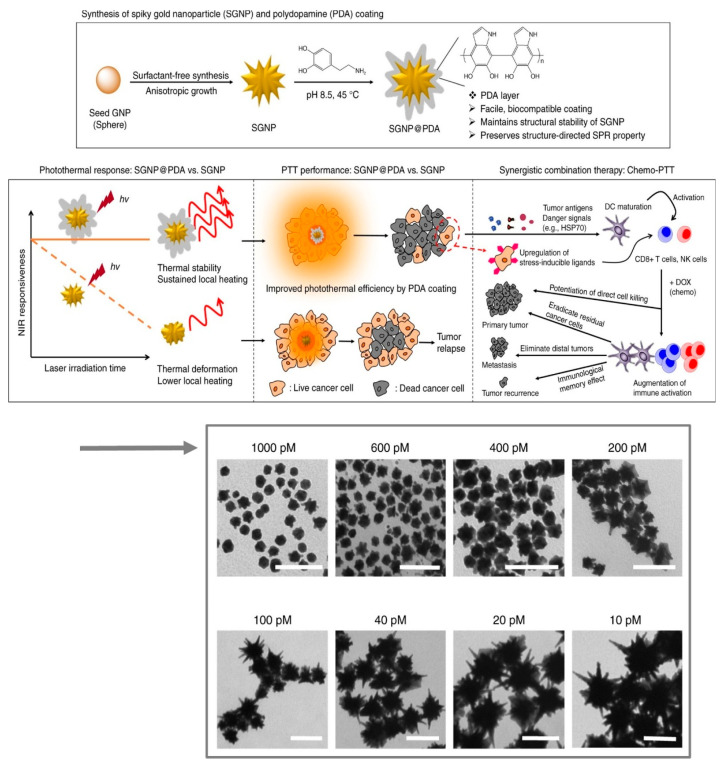
**Photothermal–Chemotherapy-Induced Antitumor Immune Activation** Polydopamine-coated spiky gold nanoparticles enhance photothermal tumor ablation and, when combined with chemotherapy, promote antitumor immune activation. Adapted from Nam et al., Nat. Commun. 9, 1074 (2018) [98], licensed under CC BY 4.0.

A single treatment is often insufficient to overcome tumor complexity and drug resistance. Therefore, combination therapy combined with different treatments is drawing attention as an important strategy. Polymeric nanosystems can deliver a variety of therapeutic substances at the same time. In addition, the drug can be selectively released in response to TME. As a result, it is used as an effective platform when combining chemotherapy with immunotherapy, phototherapy, and gene therapy [93]. Combination with immunotherapy is a treatment that activates the immune system and removes cancer cells. For example, we can consider a case of simultaneously administering a programmed death-1 (PD-1)/programmed death-ligand 1 (PD-L1) inhibitor and an anticancer drug. Tumor antigen is released by immunogenic cell death (ICD) by an anticancer drug. Furthermore, the inhibitory effect of immune checkpoints can be added to induce a strong anticancer immune response [94]. Recently, research has been underway to combine polymer nanosystems with CAR-T cell therapy and the delivery of cancer vaccines [95]. Combined with phototherapy, it includes photothermal therapy (PTT) and photodynamic therapy (PDT) (Figure 8). PTT generates heat by absorbing light of a specific wavelength by photothermal material. This heat kills cancer cells. PDT removes cancer cells through ROS generated by photosensitizer in light [96], especially PDT can induce ICD through ROS production to create synergy with immunotherapy. As a combination strategy with gene therapy, siRNA, mRNA, DNA, CRISPR-Cas9, and the like are utilized. These strategies regulate the expression of specific genes and inhibit cancer cell proliferation, drug resistance, and immune avoidance [97]. Polymeric nanosystems prevent enzymatic degradation of gene preparations. This contributes to enhanced intracellular delivery efficiency.

### 6.3. Preclinical Advances in Resistant Tumor Models

The preclinical resistant tumor model refers to a cell line or animal tumor model made to be resistant to anticancer drugs. In studies using these models, polymer nanosystems have shown excellent performance in many ways. The ability to regulate tumor accumulation, intracellular delivery chemotherapy, and drug release was observed higher than conventional anticancer drugs. The results showed improved tumor growth inhibition [99]. Based on these preclinical results, multiple polymeric nanomedicines have entered the clinical stage. Typical clinical applications include Genexol-PM and NC-6004. Genexol-PM, a Cremophor EL-free paclitaxel-loaded polymeric micelle based on PEG-b-PDLLA, has been clinically used in selected indications, including breast cancer and non-small cell lung cancer in Korea. In recurrent or metastatic breast cancer, Genexol-PM improved the objective response rate compared with conventional paclitaxel, but this improvement did not result in significant progression-free survival or overall survival benefits. This suggests that improved solubility and removal of toxic excipients alone are insufficient for clinical success [100]. NC-6004, a cisplatin-loaded PEG-poly(glutamic acid) micelle, was designed to reduce cisplatin-associated toxicity and improve tumor-targeted delivery. Although early clinical studies showed tolerability and antitumor potential, definitive clinical superiority remains uncertain, highlighting the need to align nanomedicine design with current standard regimens and indication-specific clinical needs [101,102,103]. Several other platforms received clinical evaluations. NK105, another paclitaxel-loaded polymeric micelle, was evaluated in a phase III trial for metastatic or recurrent breast cancer. Although it reduced severe peripheral neuropathy compared with conventional paclitaxel, it failed to meet the primary non-inferiority endpoint, indicating that toxicity reduction must be accompanied by clear efficacy improvement [51]. The SP1049C is a pluronic micelle with doxorubicin. CRLX101, a cyclodextrin-based polymer–camptothecin conjugate, showed promising early-phase activity. However, randomized clinical evaluation in metastatic renal cell carcinoma failed to demonstrate clear improvement in progression-free survival over standard therapy, suggesting that favorable pharmacokinetics and tumor localization do not guarantee clinical efficacy [104]. BIND-014, a PSMA-targeted docetaxel-containing polymeric nanoparticle, showed manageable toxicity, a distinct pharmacokinetic profile, and early antitumor signals in advanced solid tumors. Nevertheless, its translation was limited by target-expression heterogeneity and insufficient biomarker-driven patient selection [103,105]. Development was interrupted as some fell short of expectations. However, these cases have taught important lessons in the clinical application of polymeric nanomedicines. This reveals the clinical potential of polymeric nanomedicines and the translational challenges addressed in Section 7 below.

### 6.4. Route-Dependent Considerations for Polymeric Drug Delivery Systems

The route of administration is a key design parameter for polymeric drug delivery systems. Intravenous administration is widely used in cancer nanomedicine to allow systemic transfer to primary and metastatic tumors. However, intravenously injected nanoparticles are limited by the removal of mononuclear phagocyte systems, the accumulation in the liver and spleen, and the accumulation of heterogeneous tumors due to variable EPR effects [106]. Oral administration improves patient convenience and allows repeated administration, oral anticancer drug administration is often limited due to low solubility and permeability, gastrointestinal instability, P-gp-mediated leakage, and first-pass metabolism. Therefore, polymer nanocarriers have been studied to improve drug stability, intestinal absorption and oral bioavailability [107]. Local or intratumoral administration can increase local drug concentration and reduce systemic toxicity, but restricted penetration, high intratumor pressure, abnormal tumor substrates, and irregular intratumor distribution remain important challenges [108]. Therefore, the route of administration should be considered along with particle size, surface chemistry, carrier stability, target strategy and drug release dynamics. 

## 7. Challenges, Future Perspectives, and Conclusions

### 7.1. Heterogeneity and Translational Barriers of the Tumor Microenvironment

The factor that further complicates the therapeutic intervention of TME is heterogeneity. If the composition and function of TME were outlined in Section 2, this section focuses on the three-dimensional and preclinical model limitations of heterogeneity that hinder clinical translation. First of all, interpersonal heterogeneity refers to the significant difference in TME cell composition, vascular density, ECM hardness, and immune status for each patient, even if they are of the same histological cancer species [109]. In particular, variability in the EPR effect is a prime example. In the preclinical animal model, the effect of EPR was observed relatively consistently. However, human tumors have very different characteristics of vascular permeability, tumor vascular density, and interstitial characteristics [106]. As a result, passive accumulation strategies dependent on EPR vary greatly in reactivity for each patient. This is due in part to the fact that specific target or stimulus responsiveness designs are effective only in some patient populations, which does not show significant differences in overall clinical trials. Next, the spatial heterogeneity within the tumor refers to the fact that the vascular proximity, hypoxia, necrosis, and invasive boundary sites have different TME characteristics within the same tumor [110,111]. Therefore, the single-stimulus reactive system can function selectively only in certain areas of the tumor. This causes incomplete therapeutic responses and survival of residual cells. In addition, temporary heterogeneity reflects dynamic changes in TME before and after treatment. For example, antiangiogenic treatment temporarily normalizes blood vessels but then worsens hypoxic conditions. Immunotherapy dramatically changes the composition of immune compartments [112]. In addition, the divergence between the preclinical model and the actual patient TME is an important conversion barrier. Traditional xenograft models do not adequately reproduce human ECM and immune systems [113]. Therefore, the introduction of next-generation models such as patient-derived xenotransplantation (PDX), patient-derived organoids (PDOs), and microfluidic tumors on-chip is required. The development of single-cell RNA sequencing and spatial transcriptomics redefines the cell diversity and spatial structure of TME. Based on these factors, stratified patient selection and customized nano-platform design will be the key to clinical success in the future.

### 7.2. Safety, Scalability, and Regulatory Considerations

A polymer nano-based drug delivery system provides excellent efficiency. However, there are many challenges in terms of safety, scalability, and regulation. From a safety perspective, some macromolecules and inorganic nanomaterials can cause immune responses in the body. In particular, PEG nanoparticles can cause complement activation-related pseudoallergy (CARPA) [114]. Repeated administration results in the formation of anti-PEG antibodies and the accelerated blood clearance phenomenon. This limits the therapeutic effectiveness [115], and long-term toxicity from accumulation of nanoparticles into the liver and spleen needs to be continuously evaluated. From a scalability perspective, the clinical translation of polymeric nanomedicines is severe in each aspect of Chemistry, Manufacturing, and Control (CMC). Batch-to-batch reproducibility of polymer nanoparticles, ensuring aseptic properties, endotoxin management, and long-term storage stability are all important elements of quality control. Self-assembly-based systems may have significant changes in particle characteristics even with small changes in process parameters. Large-scale production therefore remains challenging [116]. Regulatory evaluation is also guided by major frameworks, including the U.S. FDA guidance for drug products containing nanomaterials, the EMA reflection paper on nanomedicines, and ISO/TC 229 nanotechnology standards [117,118]. Unlike general low-molecular drugs, polymeric nanomedicines require complex characterization. This includes particle size distribution, surface characteristics, release profiles, and analysis of protein corona. To this end, the development of standardized characterization protocols and biological equivalence evaluation standards is continuously required.

### 7.3. Future Directions for Precision Polymeric Nanomedicine

The future development of polymeric nanomedicines depends on the combination of the precision medicine paradigm. This means developing customized treatment strategies tailored to each patient’s tumor microenvironment. In terms of design, the development of multi-stimulus reactivity and multifunctional integration platforms has become an important direction beyond a single strategy. The single strategy includes pH reactivity, hypoxia reactivity, ligand-mediated targeting, cell membrane coating, and charge conversion systems [119]. In particular, the Theranostic nanoplatform, which combines diagnosis and treatment, enables real-time monitoring of treatment responses. Consequently, adaptive treatment adjustment can be realized. From the perspective of technology integration, machine learning (ML) and artificial intelligence (AI) are expected to accelerate the development of polymeric nanomedicines. Specifically, there are various utilization directions. First, AI-based polymer candidate screening and de novo design. Second, prediction of compatibility and loading efficiency between the drug and the polymer. Third, prediction of the composition and biological distribution of protein corona. Fourth, insilico PK/PD modeling [120] and individual screening utilizing patient-derived organoids (PDOs) and microfluidic tumor-on-chip models are drawing attention. Digital twin-based treatment simulations are evaluated as next-generation tools that can predict treatment responses before clinical application [121]. In terms of clinical translation, the design of adaptive clinical trials, patient stratification based on biomarkers, and the development of polymeric nanomedicines combined with precision diagnosis will be important directions. Such precision treatment provides the possibility of minimizing side effects and maximizing treatment effects.

## 8. Conclusions

This review discusses the TME-based drug resistance problem and comprehensively reviews the possibility and current status of a polymeric nanomedicines delivery system to overcome it. TME induces multilayer resistance at the cellular, physicochemical, and molecular levels. To overcome this, multiple mechanisms, not single targets, need to be adjusted. Polymeric nanotechnology showed broad potential in selective delivery, targeting, improving stability, and increasing treatment efficiency of drugs. Some preparations have already entered the clinical stage. However, there are still challenges to be solved, such as heterogeneity-based treatment variability, EPR effect reliability limitations, safety, scalability, and regulatory complexity. In the future, the development of precision polymeric nanomedicines combined with AI, single-cell technology, and organoid-based models has the potential to gradually expand the possibility of clinical use in specific indications. TME-reactive nanosystems have been shown to develop into an important therapeutic auxiliary axis in therapy-resistant cancer through combination with immunotherapy, phototherapy, and gene therapy.

## Figures and Tables

**Table 1:** Comparison of stimuli-responsive polymeric nanosystems for tumor-targeted drug delivery.

Stimulus	TME Characteristics	Responsive Mechanism	Key Chemical Structures	Drug Release Principle
**pH**	Tumor: pH ~6.5 Endosome: pH 5.0–6.0 Lysosome: pH 4.0–5.0 (Normal tissue: pH 7.4)	Cleavage of acid-labile bonds under acidic conditions	Hydrazone, acetal, orthoester, imine bond	Micelle dissociation → intracellular drug release
**Redox**	Intracellular GSH: ~10 mM Extracellular GSH: ~2 µM (~100–1000× higher)	Thiol–disulfide exchange by elevated intracellular GSH	Disulfide bond (–S–S–)	Reductive cleavage of disulfide bonds → nanoparticle degradation → drug release
**ROS**	Elevated ROS due to rapid metabolism and mitochondrial dysfunction	Oxidative cleavage of ROS-sensitive linkages	Thioketal, boronic ester, arylboronate, selenide	ROS-triggered bond cleavage → polymer disassembly → drug release
**Hypoxia**	O_2_ partial pressure < 10 mmHg Overexpressed nitroreductase	Reduction in hypoxia-sensitive groups, altering hydrophilic/hydrophobic balance	Azo bond, nitroimidazole (2-NI), nitrobenzene	Hydrophobic-to-hydrophilic conversion → micelle disruption → drug release

## Data Availability

No new data were created or analyzed in this study.

## Abbreviations

The following abbreviations are used in this manuscript:

TME	Tumor microenvironment
PDDSs	Polymeric drug delivery systems
CAFs	Cancer-associated fibroblasts
ECM	Extracellular matrix
IFP	Interstitial fluid pressure
EPR	Enhanced permeability and retention
GSH	Glutathione
ROS	Reactive oxygen species
TGF-β	Transforming growth factor-beta
MDSCs	Myeloid-derived suppressor cells
TAMs	Tumor-associated macrophages
BCL-2	B-cell lymphoma 2
ABC	ATP-binding cassette
MDR	Multidrug resistance
PLGA	poly(lactic-co-glycolic acid)
PEG	Polyethylene glycol
LPNs	Lipid–polymer hybrid nanoparticles
AI	Artificial intelligence
